# P-500. Awareness and preferences for every 2 month long-acting injectable HIV Pre-Exposure Prophylaxis (PrEP) versus daily oral PrEP among cisgender women in the United States and the Dominican Republic

**DOI:** 10.1093/ofid/ofae631.699

**Published:** 2025-01-29

**Authors:** Aimee A Metzner, Alan Oglesby, Allison O’Rourke, Noya Galai, Tahilin Sanchez Karver, Wendy W Davis, Rachel Scott, Patricia Moriarty, Tamara Taggart, Clare Barrington, Hoisex Gomez, Martha Perez, Yeycy Donastorg, Deanna Kerrigan

**Affiliations:** ViiV Healthcare, Durham, North Carolina; ViiV Healthcare, Durham, North Carolina; George Washington University, Washington, District of Columbia; John Hopkins Bloomberg School of Public Health, Baltimore, Maryland; Johns Hopkins University, Baltimore, MD; George Washington University Milken Institute School of Public Health, Washington, District of Columbia; MedStar Health Research Institute, Washington, DC; MedStar Washington Hospital Center, Washington DC, District of Columbia; George Washington University, Washington, District of Columbia; UNC Gillings School of Global Public Health, Chapel Hill, North Carolina; Unidad de Vacunas e Investigación IDCP, Santo Domingo, Distrito Nacional, Dominican Republic; Instituto Dermatologico y Cirugia de la Piel, Santo Domingo, Distrito Nacional, Dominican Republic; INSTITUTO DERMATOLOGICO Y CIRUGIA DE PIEL "DR. HUBERTO BOGAERT DIAZ", SANTO DOMINGO, Distrito Nacional, Dominican Republic; George Washington University, Washington, District of Columbia

## Abstract

**Background:**

Globally, cisgender women (CGW) represent 53% of people with HIV and 45% of new acquisitions (2022). Female sex workers (FSW) are 30 times more likely to acquire HIV (vs women overall); in the US 54% of new diagnoses in CGW are Black women. HIV pre-exposure prophylaxis (PrEP) coverage in US CGW remains low at 15%. Long-acting injectable (LAI) PrEP may help address inequities and expand use, as 2013-22 CDC data shows 7% of US people prescribed oral PrEP were female compared to 12.5% for injectable PrEP. Presented here is a mixed methods study assessing awareness and preferences for PrEP in CGW.

**Figure:**
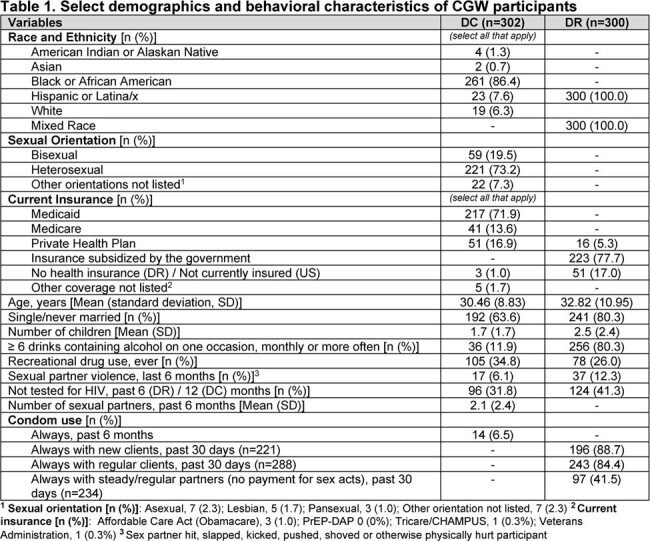

**Methods:**

Cross-sectional surveys were conducted among CGW in Washington, DC and Santo Domingo, Dominican Republic (DR) from 11/’22-07/’23. DC CGW were recruited in reproductive health clinics; DR CGW were FSW recruited by peer navigators. CGW were ≥ 18 years, receiving care at the participating clinic and not living with HIV.

**Figure:**
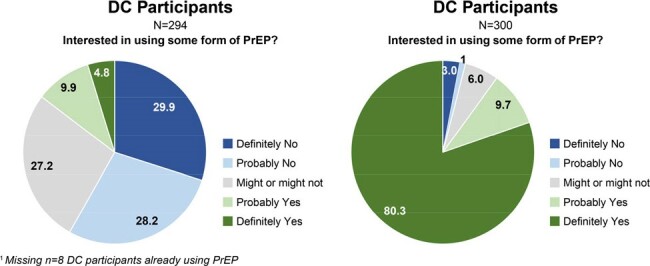

**Results:**

In DC (n=302), 86.4% identified as Black; in the DR (n=300), all identified as mixed race and Latina ethnicity. 71.2% (DC) and 94.3% (DR) never heard of oral PrEP; 91.1% (DC) and 98.3% (DR) never heard of LAI PrEP. 3% (DC) and 1% (DR) had ever used PrEP. Mean number of sex partners (past 6 months, DC) was 2.1, with 6.5% always using a condom [Table 1]. In the DR, always condom use was > 80% for sex with clients, < 42% with steady partners. An interest in using some form of PrEP was 4.8% in DC vs 80.3% in the DR [Figure 1]. Preference for LAI PrEP was 73.2% (DC) and 71% (DR) among all participants [Figure 2]. CGW thought it easier to get to a clinic every 2 months than to take a daily pill (DC, 64.9%; DR, 60.0%;) [Table 2], and would worry more about people discovering PrEP pills than being seen at a PrEP clinic for injections (DC, 59.9%; DR, 55.7%). PrEP service location preferences were family planning or primary care clinics (DC), and pharmacies or sexual health clinics (DR). Self-assessment of potential HIV acquisition was majority not likely/impossible (DC, 88%; DR, 53%); most DC CGW never used PrEP because they had not heard of it.

**Figure:**
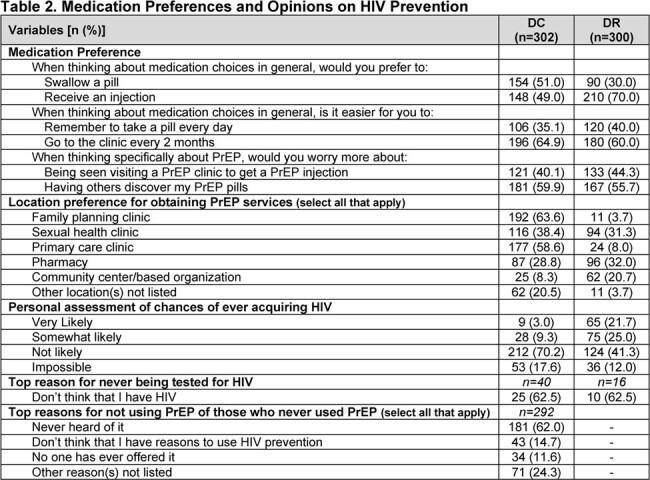

**Conclusion:**

Gaps in HIV knowledge, PrEP awareness and uptake exist for CGW. PrEP decision making is multifactorial and varies by population. CGW should be informed of their options and educated about potential reasons for PrEP use in a setting accessible and comfortable to them.

**Figure:**
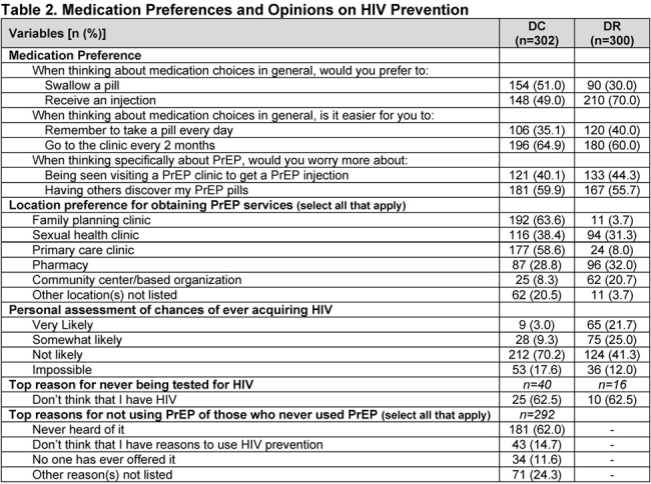

**Disclosures:**

**Aimee A. Metzner, PharmD, AAHIVP**, GSK: Stocks/Bonds (Public Company)|ViiV Healthcare: Employee **Alan Oglesby, MPH**, GSK: Stocks/Bonds (Public Company)|ViiV Healthcare: Employee **Allison O’Rourke, MPH**, ViiV Healthcare: Grant/Research Support **Wendy W. Davis, EdM**, ViiV Healthcare: Grant/Research Support **Rachel Scott, MD,MPH,FACOG**, DHHS Perinatal Guidelines: Board Member|UW STD Prevention Training Center (UW STD PTC): Honoraria|ViiV Healthcare: Advisor/Consultant|ViiV Healthcare: Grant/Research Support|Vindico CME: Honoraria **Tamara Taggart, PhD, MPH**, HealthHIV: Honoraria **Deanna Kerrigan, PhD, MPH**, ViiV Healthcare: Grant/Research Support

